# Overlooked and Underdiagnosed: Cardiac Sarcoidosis and Unexplained Ventricular Tachycardia and Fibrillation

**DOI:** 10.1016/j.jacadv.2025.102246

**Published:** 2025-10-27

**Authors:** Aaron A. Sifuentes, Ghazaleh Goldar, Usamah M. ElBakkush, Kaushik Gokul, Mohammed Mhanna, Peter Farjo, Paari Dominic

**Affiliations:** a Division of Cardiovascular Medicine, University of Iowa Hospitals and Clinics, Iowa City, Iowa, USA; b Department of Internal Medicine, Wake Forest University School of Medicine, Winston-Salem, North Carolina, USA; c Department of Internal Medicine, University of Iowa Hospitals and Clinics, Iowa City, Iowa, USA

**Keywords:** sarcoidosis, ventricular fibrillation, ventricular tachycardia

## Abstract

**Background:**

Cardiac sarcoidosis (CS) often manifests as unexplained ventricular tachycardia (VT) and ventricular fibrillation (VF) but remains significantly underdiagnosed. Although guidelines recommend advanced diagnostics, many clinicians may not pursue this in patients presenting with unexplained VT/VF.

**Objectives:**

The objective of the study was to assess the proportion of patients with unexplained VT/VF who underwent guideline-based evaluation for CS.

**Methods:**

Using TriNetX data, we conducted a retrospective cohort study of patients aged 18 years or older who presented with unexplained VT/VF requiring implantable cardioverter defibrillator device implantation over the past 5 years. We evaluated the use of cardiac diagnostic modalities; including cardiac magnetic resonance imaging, positron emission tomography imaging, chest computed tomography, and myocardial biopsy, and monitored subsequent diagnoses of CS over a 5-year follow-up period.

**Results:**

Of 732 patients with VT/VF across 56 healthcare organizations, the mean age was 53 ± 19 years. Of these, 61% were male and 73% were White, while 8% were African American. The mean follow-up duration was 827 days (interquartile range: 0-1,1167). Cardiac magnetic resonance imaging was performed in 146 patients (19.9%; 95% CI: 17.21%-22.99%), cardiac positron emission tomography in 17 patients (2.3%; 95% CI: 1.45%-3.69%), chest computed tomography in 54 patents (7.4%; 95% CI: 5.70%-9.50%), and myocardial biopsy in < 10 patients (1.4%; 95% CI: 0.74%-2.50%). Sarcoidosis was diagnosed in <10 patients (1.4%; 95% CI: 0.74%-2.50%).

**Conclusions:**

Despite established guidelines, testing for CS remains underutilized in patients with unexplained VT/VF. This diagnostic gap contributes significantly to the lower prevalence of CS (1.4%) than the expected 17% to 28% in this cohort and suggests an area for improvement.

Sarcoidosis is a multisystem granulomatous disorder that can lead to life-threatening conditions, including high-grade atrioventricular block, ventricular arrhythmias, and heart failure.^1^, ^2^, ^3^ Cardiac involvement, the leading cause of death related to sarcoidosis, is clinically apparent in only 5% of patients with sarcoidosis, although myocardial lesions are detected at autopsy in 20% to 60% of cases.^4^ Per prior reports approximately 17% to 28% of patients with unexplained ventricular tachycardia (VT) or ventricular fibrillation (VF) potentially have underlying cardiac sarcoidosis (CS), yet it is often missed in clinical practice.^1^^,^^3^^,^^5^

Diagnosing CS can be challenging due to nonspecific symptoms and overlap with other cardiac conditions, such as ischemic heart disease and myocarditis.^1^^,^^2^ Moreover, the standard diagnostic tools such as echocardiography are often insufficient to detect the subtle myocardial involvement seen in CS.^2^ Recent advancements in cardiac imaging, particularly cardiac magnetic resonance imaging (cMRI) and positron emission tomography (PET), have greatly improved the ability to detect CS in its early stages. Chest computed tomography (CT), although not part of the guideline recommended workup, can provide insight into potential extra-CS, especially at centers where more advanced imaging techniques are unavailable. Myocardial biopsy is the gold standard for confirming CS by visualizing noncaseating granulomas.^1^^,^^2^ However, this is often reserved for cases of inconclusive imaging, due to its invasiveness and associated risks.^2^ Even with biopsy, sensitivity is low at 20% due to the patchy nature of the disease and the potential sampling of unaffected areas. However, sensitivity can increase to 40% to 50% when guided by voltage mapping.^2^ The American Heart Association recommends considering CS in patients with unexplained VT/VF, particularly when other causes of VT/VF have been excluded.^2^ These guidelines emphasize the importance of immunosuppressive/antiarrhythmic therapy to prevent progression and cardiac complications.^1^, ^2^, ^3^ Treatment may enhance conduction in cases of atrioventricular block, but their effects on ventricular arrhythmias, left ventricular dysfunction, and mortality are not yet firmly established.^2^ Treatment of the latter 2 is inferred from smaller studies that show left ventricular dysfunction, and ventricular arrhythmias respond to treatment, but there is currently a lack of robust high-powered studies.^2^^,^^3^^,^^6^, ^7^, ^8^, ^9^ Although clear guidelines exist, a substantial disconnect is believed to persist between clinical recommendations and real-world practice. This study aims to assess the use of diagnostic testing for CS in unexplained VT/VF.

## Methods

### Study design

This study is a retrospective cohort analysis seeking to identify the number of patients presenting with unexplained VT/VF who received guideline-recommended workup of CS.

### Data source

This study used data from the TriNetX Global Health Research Network (TriNetX, Inc), a federated health data platform that aggregates deidentified electronic health records from over 90 health care organizations (HCOs) across the world. The TriNetX platform provides access to data on patient demographics, diagnoses, procedures, medications, and outcomes, allowing for large-scale epidemiological analyses. As a federated network, TriNetX received a waiver from Western Institutional Review Board as only aggregated counts and statistical summaries of deidentified information are received, no protected health information is received, and no study-specific activities are performed in retrospective analyses. This query was run in May 2025 on the network research (with natural language processing) with data provided from 56 HCOs. The natural language processing functionality was available within the network but was not usede for patient selection or outcome assessment in this analysis. Our study relied exclusively on structured data derived from International Classification of Diseases, Tenth Revision (ICD-10) and Current Procedural Terminology (CPT) codes.

### Patient selection

We used the ICD-10 diagnosis and procedure codes, and the Centers for Medicare and Medicaid CPT codes (Supplemental Table 1) to identify patients. This study included patients aged ≥18 years who were diagnosed with unexplained VT/VF necessitating placement of an implantable cardioverter defibrillator within 30 days, followed over a 5-year period (2020-2025). Unexplained VT/VF was defined as VT/VF without any known underlying cause. For the inclusion and exclusion criteria please see Table 1.

**Table 1:** Inclusion and Exclusion Criteria

Inclusion criteria
Age ≥18
Diagnosis of VT or VF
Implantable cardioverter-defibrillator device insertion within 30 days of being diagnosed with VT or VF
Exclusion criteria
Previously diagnosed sarcoidosis or sarcoid myocarditis
Acute myocardial infarction
Ischemic heart diseases
Cardiomyopathy (eg, dilated cardiomyopathy, hypertrophic cardiomyopathy, restrictive cardiomyopathy, substance induced cardiomyopathy)
Long QT syndrome
Other cardiac ion channelopathies (eg, Brugada syndrome, long QT syndrome, short QT syndrome catecholaminergic polymorphic ventricular tachycardia (CPVT))
Left ventricular ejection fraction ≤35%
Prior diagnosis of amyloidosis
Pre-excitation/accessory pathway syndromes
Thyrotoxicosis (thyrotoxic crisis or storm)
Infective myocarditis

VF = ventricular fibrillation; VT = ventricular tachycardia.

### Index event

In our study, the index event was defined as the date on which a patient was diagnosed with VT/VF.

### Time window

Our analysis started on the first day of the index event and continued until 5 years after the first occurrence of the index event. Our cohort includes patients between the years 2020 and 2025.

### Outcome

The primary outcome measure was the proportion of patients who underwent diagnostic testing for CS, including cMRI, PET scans, chest CT, and myocardial biopsy. The secondary outcome measure was the diagnosis of sarcoidosis during the follow-up period. Cardiac MRI, PET scan, chest CT, myocardial biopsy, and sarcoidosis diagnosis were identified using CPT codes shown in Supplemental Table 1.

### Statistical analysis

Analyses were conducted using the TriNetX Analytics platform and no external statistical software was used. Continuous variables were summarized as mean ± SD or median with IQR, and categorical variables as counts and percentages. For proportions, 95% CIs were calculated using the Wilson score method, the default approach within TriNetX. Analyses were descriptive in nature; no propensity score matching or hypothesis testing was performed.

## Results

TriNetX identified 732 patients diagnosed with VT/VF which met the inclusion and exclusion criteria across 56 HCOs. Among them, 534 patients (73%) were from academic centers (38 HCOs). Baseline clinical characteristics and geographical details of the cohort are summarized in Tables 2 and 3, respectively. The mean age of patients was 53 ± 19 years, with an age range of 18 to 90, 61% were male and 73% were White, whereas 8% were African American. Over a median follow-up of 827 days (IQR: 0-1,167 days), cMRI was performed in 146 patients (19.9%; 95% CI: 17.21%-22.99%), cardiac PET in 17 patients (2.3%; 95% CI: 1.45%-3.69%), chest CT in 54 patents (7.4%; 95% CI: 5.70%-9.50%), and myocardial biopsy in < 10 patients (1.4%; 95% CI: 0.74%-2.50%). Overall, only 26% of patients underwent advanced testing (MRI, PET, chest CT, or biopsy), with an ultimate CS diagnosis rate of ≤1.4% (95% CI: 0.74%-2.50%) (Table 4). Characteristics and geographical details specific to patients who underwent any of the diagnostic evaluations and those without any evaluation are detailed in Table 5. Study design and results are demonstrated in the Central Illustration.

**Table 2:** Baseline Characteristics of the Cohort (N = 732)

Age (y)	53 ± 19
Sex	
Male	446 (61%)
Female	278 (38%)
Ethnicity	
White	534 (73%)
Black	58 (8%)
Asian	29 (4%)
American Indian or Alaska	10 (1.4%)
Native Hawaiian or other Pacific Islander	10 (1.4%)
Other or unknown race	95 (13%)
Comorbid conditions	
SVT	190 (26%)
Essential (primary) hypertension	322 (44%)
Atrial fibrillation or flutter	271 (37%)
First degree atrioventricular block	80 (11%)
Advanced heart block	124 (17%)
Premature ventricular contractions	248 (34%)
Premature atrial contractions	80 (11%)
Left bundle branch block	44 (6%)
Right bundle branch block	139 (19%)
Sick sinus syndrome	102 (14%)
Type 2 diabetes mellitus	95 (13%)
Chronic kidney disease	51 (7%)

Values are mean ± SD or n (%).

SVT = supraventricular tachycardia.

**Table 3:** Geographic Distribution of Patients in the Cohort (N = 732)

Northeast	273 (37%)
Midwest	95 (13%)
South	229 (31%)
West	117 (16%)
Unknown	18 (2%)

Values are n (%).

**Table 4:** Summary of Prevalence of Outcomes (N = 732)

cMRI	146 (19.9%) [95% CI: 17.21–22.99]
Cardiac PET	17 (2.3%) [95% CI: 1.45–3.69]
Myocardial biopsy	≤10∗ (≤1.4%) [95% CI: 0.74–2.50]
Patient with at least 1 of the following: cMRI, cardiac PET, myocardial biopsy	158 (21.6%) [95% CI: 18.76–24.71]
Chest CT	54 (7.4%) [95% CI: 5.70–9.50]
Any of: cMRI, cardiac PET, biopsy, or chest CT	194 (26.5%∗) [95% CI: 23.43–29.82]
Diagnosis of sarcoidosis	≤10∗ (≤1.4%) [95% CI: 0.74–2.50]

cMRI = cardiac magnetic resonance imaging; CT = computed tomography; PET = positron emission tomography.

∗ To protect patient privacy, numbers are rounded up to 10 in the TriNetX database.

**Table 5:** Characteristics and Geographic Distribution of Patients Who Did Versus Did Not Undergo Any Diagnostic Evaluation

	Patients With at Least 1 Diagnostic Testing (n = 171)	Patients Without Any Diagnostic Evaluation (n = 557)	*P* Value
Characteristics			
Age (years)	51 ± 18	53 ± 19	<0.001
Sex			
Male	111 (65%)	334 (60%)	0.284
Female	56 (33%)	212 (38%)	0.242
Unknown	4 (2%)	11 (2%)	1.000
Ethnicity			
White	126 (74%)	401 (72%)	0.738
Black	17 (10%)	45 (8%)	0.544
Asian	8 (5%)	22 (4%)	0.842
American Indian or Alaska	0 (0%)	11 (2%)	0.135
Native Hawaiian or other Pacific Islander	8 (5%)	11 (2%)	0.096
Other or unknown race	10 (6%)	67 (12%)	0.031
U.S. region			
Northeast	61 (36%)	200 (36%)	0.895
Midwest	31 (18%)	73 (13%)	0.129
South	44 (26%)	178 (32%)	0.247
West	25 (15%)	95 (17%)	0.787
Unknown	10 (6%)	11 (2%)	0.017
Comorbid conditions			
SVT	51 (30%)	134 (24%)	0.157
Essential (primary) hypertension	82 (48%)	245 (44%)	0.410
Atrial fibrillation or flutter	60 (35%)	212 (38%)	0.540
First degree atrioventricular block	22 (13%)	61 (11%)	0.581
Advanced heart block	29 (17%)	95 (17%)	1.00
Premature ventricular contractions	68 (40%)	178 (32%)	0.072
Premature atrial contractions	22 (13%)	58 (10%)	0.449
Left bundle branch block	8 (5%)	33 (6%)	0.668
Right bundle branch block	39 (23%)	106 (19%)	0.331
Sick sinus syndrome	18 (11%)	83 (15%)	0.186
Type 2 diabetes mellitus	32 (19%)	61 (11%)	0.011
Chronic kidney disease	12 (7%)	39 (7%)	1.000

Abbreviations as in Table 2.

## Discussion

Our findings indicate that only a small proportion of patients presenting with unexplained VT/VF underwent evaluation for CS, which is inconsistent with the major clinical guidelines.^1^^,^^2^ Ultimately around 1.4% of patients in our cohort were diagnosed with sarcoidosis, a rate much lower than the reported prevalence of CS in patients with unexplained VT/VF. A review by Ribeiro Neto et al. estimated that the prevalence of CS in patients presenting with unexplained VT/VF was between 17% to 28%.^1^ The lower-than-expected prevalence of sarcoidosis in our study may, in part, reflect the racial composition of our cohort, as sarcoidosis is more common in African American populations and our study population was predominantly White. In addition, the low diagnostic yield seen in this cohort is likely not a reflection of true disease prevalence, but suggests possible gaps in the diagnostic process, based on the low rate of CS evaluations seen in our study.

Beyond CS, our findings highlight a broader issue in the evaluation of patients with unexplained VT/VF. Many patients did not undergo any diagnostic testing, suggesting that underevaluation may lead to missed diagnoses across a spectrum of potentially serious cardiac conditions beyond sarcoidosis. This underscores the importance of implementing systematic and comprehensive diagnostic pathways to ensure that all possible underlying causes are considered.

The demands of busy clinical practice may restrict the ability to prioritize the necessary diagnostic steps for evaluating CS. The nonspecific nature of CS symptoms makes it difficult to diagnose without specialized testing. Although patients with CS may present with symptoms such as syncope, palpitations, nonspecific conduction abnormalities, or heart failure, these symptoms are more commonly attributed to other cardiac conditions. When cardiac dysfunction is the only manifestation of sarcoidosis, the diagnosis is seldom considered.^5^ The diagnostic workup of CS relies on advanced imaging techniques, such as cMRI and PET, which are expensive and may not be accessible in all clinical settings, especially in resource-limited areas.^5^ Furthermore, cMRI is generally contraindicated within the first 6 weeks following device placement. In addition, although myocardial biopsy is considered the gold standard for diagnosing CS, it is an invasive procedure that carries inherent risks and is generally reserved for cases where imaging results are inconclusive.^1^^,^^2^ This hesitancy to perform invasive tests may contribute to the low diagnostic rates observed in this study. In addition, given the accessibility challenges associated with guideline-recommended diagnostics such as cMRI and PET, we also explored the use of chest CT, even though it is not formally included in the recommended workup for CS. Nevertheless, only 7.4% of patients underwent chest CT, highlighting a persistent underutilization of even more readily available imaging modalities. Although these barriers may not indicate a lack of awareness, they can contribute to the underdiagnosis of CS in patients presenting with unexplained VT/VF.

The consequences of failing to diagnose CS are substantial, as untreated CS patients face an elevated risk of life-threatening arrhythmias, progressive heart failure, and sudden cardiac death.^1^, ^2^, ^3^^,^^5^ A study conducted by Ekström et al. showed that sudden cardiac death, including both fatal and aborted cases, accounts for approximately 11% to 14% of the presenting manifestations of CS.^10^^,^^11^ A study from Padala et al. showed early treatment with immunosuppressive therapies may improve outcomes in VT and left ventricular dysfunction, further highlighting the clinical importance of maintaining a high index of suspicion for evaluating CS in patients presenting with unexplained VT/VF.^8^ These findings highlight the need to improve clinician awareness of diagnostic indicators and ensure early referral to appropriately equipped centers for timely evaluation and management of suspected CS.

### Strengths

The use of the TriNetX research network enabled access to a large, geographically diverse cohort of patients across 56 HCOs, enhancing the generalizability of this study’s findings. This study focused specifically on patients presenting with unexplained VT/VF, a critical clinical scenario where timely identification of underlying CS is essential. By targeting this high-risk subset, the study addresses a key knowledge gap regarding the prevalence of guideline-recommended diagnostic evaluations for CS.

### Study Limitations

As a retrospective observational study, it is inherently prone to biases in data collection and interpretation, including the potential for selection bias. Furthermore, the TriNetX database primarily reflects data from the United States, potentially limiting the external validity of our findings to populations or health care systems outside this region. The study uses ICD-10 and CPT codes for patient identification and outcome assessment, making the quality and completeness of the data dependent on the accuracy of coding practices. Any inaccuracies or omissions in coding could lead to bias or misclassification. To protect patient privacy, numbers are rounded up to 10 in the TriNetX database and therefore exact number of patients in those groups are unclear. This may impact results, particularly for small cohorts and infrequent outcomes. Another very important limitation is that although CS was identified in a small portion of patients (1.4%); the diagnostic method could not be verified, as the TriNetX database lacks patient-level clinical detail due to privacy constraints. In addition, although prior studies have established that CS, particularly as the initial or sole manifestation, is associated with poor outcomes, we were unable to analyze long-term clinical outcomes for the small subset of patients diagnosed with CS due to the inherent limitations of the TriNetX database, which does not provide detailed longitudinal follow-up for rare subgroups.

## Conclusions

CS remains significantly underdiagnosed in patients presenting with unexplained VT/VF, as highlighted by the notably low utilization of advanced diagnostic testing observed in this study. This underdiagnosis carries significant risks, including delayed treatment and the potential for adverse clinical outcomes such as heart failure or sudden cardiac death. Bridging this diagnostic gap with greater clinician awareness and guideline adherence is the key for timely and appropriate diagnostic testing for CS, particularly in younger patients with unexplained VT/VF. Future studies are needed to further identify underlying barriers to guideline adherence and to explore strategies for improving the diagnosis and management of CS, with the goal of improving care and outcomes for this high-risk population.

Perspectives **COMPETENCY IN MEDICAL KNOWLEDGE:** CS should be considered in all patients with unexplained VT/VF, and clinicians should use appropriate imaging techniques, such as MRI, PET, or biopsy, to enhance diagnostic accuracy. A high degree of clinical suspicion and timely diagnosis of CS is essential, as it directly influences management decisions and improves patient outcomes, reducing the risk of cardiac complications.

**TRANSLATIONAL OUTLOOK:** This study highlights the low rate of diagnostic testing for CS in patients with unexplained VT/VF, with only a small fraction undergoing cardiac MRI, PET scans, or myocardial biopsy. This study is the first, to our knowledge, to report on the prevalence of sarcoidosis diagnosis in this population, revealing that it is significantly underdiagnosed, with only 1.4% of patients receiving a diagnosis, far below the expected prevalence of 17% to 28%.

**Central Illustration:**
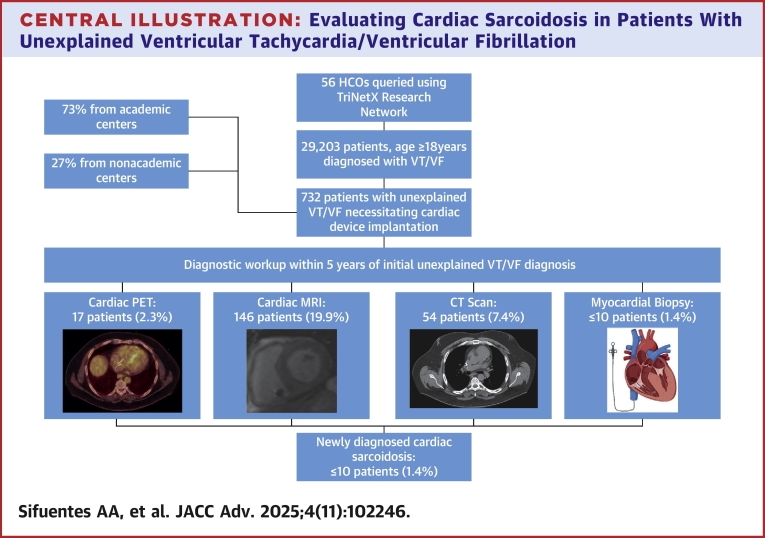
Evaluating Cardiac Sarcoidosis in Patients With Unexplained Ventricular Tachycardia/Ventricular Fibrillation

## Funding support and author disclosures

The authors have reported that they have no relationships relevant to the contents of this paper to disclose.
