# Supplementary material for: Overlooked and Underdiagnosed: Cardiac Sarcoidosis and Unexplained Ventricular Tachycardia and Fibrillation
Source: JACC Adv. 2025 Oct 27;4(11):102246. doi: 10.1016/j.jacadv.2025.102246 (PMC12596564; doi:10.1016/j.jacadv.2025.102246)
Supplement: Supplemental Material [file mmc1.docx]

**Supplementary Index**

**Supplementary Table 1: List of ICD-10-CM and CPT codes. (Intended for publication)**

| **Variable** | **ICD-10-CM or CPT Code(s)** | |
| --- | --- | --- |
|  | **Diagnosis Code** | **Procedural Code** |

| **Inclusion Criteria** | | |
| --- | --- | --- |
| Ventricular tachycardia | UMLS:ICD10CM:I47.2 |  |
| Ventricular fibrillation | UMLS:ICD10CM:I49.01 |  |
| Insertion or replacement of permanent subcutaneous implantable defibrillator system, with subcutaneous electrode, including defibrillation threshold evaluation, induction of arrhythmia, evaluation of sensing for arrhythmia termination, and programming or reprogramming of sensing or therapeutic parameters, when performed |  | UMLS:CPT:33270 |
| Insertion or replacement of permanent implantable defibrillator system, with transvenous lead(s), single or dual chamber |  | UMLS:CPT:33249 |
| **Exclusion Criteria** | | |
| Acute myocardial infarction | UMLS:ICD10CM:I21 |  |
| Ischemic heart diseases | UMLS:ICD10CM:I20-I25 |  |
| Acute myocarditis | UMLS:ICD10CM:I40 |  |
| Long QT syndrome | UMLS:ICD10CM:I45.81 |  |
| Cardiac ion channelopathies (e.g., Brugada syndrome, long QT syndrome, short QT syndrome, catecholaminergic polymorphic ventricular tachycardia) | UMLS:CPT:1029707 |  |
| Pre-excitation syndrome | UMLS:ICD10CM:I45.6 |  |
| Amyloidosis | UMLS:ICD10CM:E85 |  |
| Thyrotoxicosis with toxic multinodular goiter with thyrotoxic crisis or storm | UMLS:ICD10CM:E05.21 |  |
| Dilated cardiomyopathy | UMLS:ICD10CM:I42.0 |  |
| Hypertrophic cardiomyopathy | UMLS:ICD9CM:425.1 |  |
| Obstructive hypertrophic cardiomyopathy | UMLS:ICD10CM:I42.1 |  |
| Other hypertrophic cardiomyopathy | UMLS:ICD10CM:I42.2 |  |
| Endomyocardial (eosinophilic) disease | UMLS:ICD10CM:I42.3 |  |
| Endocardial fibroelastosis | UMLS:ICD10CM:I42.4 |  |
| Other restrictive cardiomyopathy | UMLS:ICD10CM:I42.5 |  |
| left ventricular ejection fraction ≤35% ever | TNX Curated 2003 |  |
| Alcoholic cardiomyopathy | UMLS:ICD10CM:I42.6 |  |
| Cardiomyopathy due to drug and external agent | UMLS:ICD10CM:I42.7 |  |
| Other cardiomyopathies | UMLS:ICD10CM:I42.8 |  |
| Cardiomyopathy, unspecified | UMLS:ICD10CM:I42.9 |  |
| Cardiomyopathy in diseases classified elsewhere | UMLS:ICD10CM:I43 |  |
| Sarcoidosis | UMLS:ICD10CM:D86 |  |
| **Outcomes** | | |
| PET |  | UMLS:CPT:78432, UMLS:CPT:78433  UMLS:CPT:78431  UMLS:CPT:78815  UMLS:CPT:78816  UMLS:CPT:A9552  UMLS:HCPCS: A9552 |
| cMRI |  | UMLS:CPT:75561,  UMLS:CPT75563 |
| Myocardial Biopsy |  | UMLS:CPT:93505 |
| CT chest |  | UMLS:CPT:71250  UMLS:CPT:71260  UMLS:CPT:71270  UMLS:ICD10PCS:BW24  UMLS:ICD10PCS:BW25 |
| Sarcoidosis | UMLS:ICD10CM:D86 |  |
